# A novel mRNA-based therapeutic vaccine elicits robust anti-tumor immunity against HPV-associated malignancies

**DOI:** 10.3389/fimmu.2026.1823374

**Published:** 2026-04-17

**Authors:** Qi Li, Yongzhuang Liu, Yi Wang, Zhigang Li

**Affiliations:** Department of Research and Development, Liverna Therapeutics Inc., Zhuhai, China

**Keywords:** HPV, HPV-associated cancer, T cell co-stimulatory molecules, therapeutic mRNA vaccine, TriStim-E6/E7 mRNA

## Abstract

**Background:**

Human papillomavirus (HPV) infection is strongly associated with multiple malignancies, primarily driven by the viral oncoproteins E6 and E7, which play a central role in HPV-induced malignant transformation. Although current prophylactic HPV vaccines have shown remarkable efficacy in preventing initial infections, there remains an urgent need for therapeutic vaccines targeting pre-existing HPV infections and HPV-associated malignancies.

**Methods:**

In this study, we developed TriStim-E6/E7, a novel HPV mRNA vaccine that combines E6/E7 antigens with three T cell co-stimulatory molecules (CD80, 4-1BBL, and CD70). The TriStim-E6/E7 mRNA was encapsulated in lipid nanoparticles (LNPs) and administered intramuscularly to mice, followed by analysis of cellular immunogenicity. The anti-tumor efficacy of TriStim-E6/E7 mRNA was evaluated using an HPV-positive TC-1 tumor model. *In vitro* T cell activation and proliferation assays were conducted to investigate the mechanistic underpinnings of the vaccine. Additionally, safety was assessed in a PBMC-reconstituted mouse model.

**Results:**

Administration of TriStim-E6/E7 mRNA elicited robust antigen-specific cellular immune responses and achieved complete regression of HPV-positive TC-1 tumors in a syngeneic mouse model. The vaccine also demonstrated durable immunoprotection, preventing tumor recurrence upon rechallenge. Through targeted T cell depletion experiments, we established that CD8^+^ T cells are indispensable for the vaccine’s anti-tumor activity, whereas CD4^+^ T cell depletion had no significant impact on therapeutic outcomes. Treatment with TriStim-E6/E7 mRNA significantly induced the proliferation and tumor infiltration of E7 antigen-specific T cells. Mechanistic investigations revealed that TriStim-E6/E7 mRNA significantly enhanced T cell activation and proliferation *in vitro* compared to control mRNAs lacking co-stimulatory elements. Furthermore, combination therapy with anti-PD-L1 antibody synergistically enhanced the anti-tumor efficacy of TriStim-E6/E7 mRNA. To evaluate clinical translatability, we developed a humanized version (hTriStim-E6/E7) encoding human CD80, 4-1BBL, and CD70 co-stimulatory molecules together with HPV E6/E7 fusion proteins. This humanized construct effectively induced antigen-specific cellular immune responses and suppressed tumor growth in CD28/4-1BB/CD27 triple-humanized mice. Additionally, a “cytokine release storm” experiment using PBMC-reconstituted mice confirmed a favorable safety profile of TriStim-E6/E7 mRNA.

**Conclusions:**

These findings collectively demonstrate the promising therapeutic potential of TriStim−E6/E7 mRNA for clinical translation against HPV−associated malignancies.

## 1 Introduction

Human papillomavirus (HPV) infections are associated with up to 5% of all human cancer cases, and more than 99% of cervical carcinomas are attributable to HPV infection (1). Additionally, subsets of vulvar, anal, penile, vaginal, and head and neck cancers are causatively associated with HPV infection (2). There are over 200 different genotypes of HPV, some of which are classified as high-risk types due to their strong association with cancer or precancerous lesions (3). HPV16, one of the high-risk types, is detected in more than 60% of cervical cancers (4).

Prophylactic HPV vaccines, such as Gardasil and Cervarix, effectively prevent viral infection by inducing neutralizing antibodies against the HPV capsid protein L1 (5). Therapeutic HPV vaccines targeting the oncoproteins E6 and E7 have demonstrated anti-tumor activity by inducing systemic antigen-specific CD8^+^ T cell responses (6). Multiple platforms have been explored for therapeutic vaccine development, including recombinant proteins, antigen-derived peptides, cell-based vaccines, viral vector-delivered vaccines, DNA vaccines, and RNA vaccines (7, 8). Among these technologies, mRNA-LNP has emerged as an attractive platform for vaccine development due to its advantages in safety, efficacy, and rapid production (9). Indeed, several therapeutic HPV mRNA vaccines targeting E6 and E7 have been developed, showing robust *in vivo* anti-tumor efficacy and promising clinical outcomes (6). However, none of them has been clinically approved yet.

A central goal of therapeutic cancer vaccines is to induce potent and durable antigen-specific immune responses, particularly given that many patients exhibit immune tolerance and face a complex and immunosuppressive tumor microenvironment (10, 11). Adjuvants can enhance the immunogenicity of vaccines by improving antigen presentation and processing (12). Professional antigen-presenting cells, which express high levels of T cell co-stimulatory molecules, have been reported to be the most potent stimulators for the activation, differentiation, and proliferation of antigen-specific T cells (13). Therefore, incorporating T cell co-stimulatory molecules can enhance the immunogenicity of antigens. CD80 and CD86 are well-characterized stimulatory molecules that activate CD28 signaling upon TCR engagement, resulting in T cell proliferation, increased IL-2 production, and survival (14–16). 4-1BBL and CD70 are also T cell co-stimulatory molecules that activate 4-1BB and CD27 signaling, respectively (13). In addition to their roles in T cell activation and proliferation, 4-1BBL has been shown to augment suboptimal cytotoxic T lymphocytes (CTL) responses (17), while CD70 stimulation is required for the generation and long-term maintenance of T cell immunity (18).

In this study, we developed TriStim-E6/E7, a novel HPV mRNA vaccine that encodes the E6 and E7 antigens of HPV16, along with three T cell co-stimulatory molecules: CD80, 4-1BBL, and CD70. TriStim-E6/E7 mRNA induced robust antigen-specific cellular immune responses and significantly inhibited the growth of HPV-positive TC-1 tumors in a syngeneic mouse model. The vaccine also promoted the proliferation and tumor infiltration of E7-specific T cells *in vivo* and significantly enhanced T cell activation and proliferation *in vitro*. A humanized version of TriStim-E6/E7 induced antigen-specific cellular immune responses and suppressed tumor growth in CD28/4-1BB/CD27 triple-humanized mice. Additionally, TriStim-E6/E7 mRNA did not induce a “cytokine release storm” in an immune system-humanized mouse model, confirming its favorable safety profile. These findings collectively demonstrate the therapeutic potential of TriStim-E6/E7 for clinical application. Based on the preclinical data presented here, we propose that this mRNA vaccine represents a promising strategy for the treatment of HPV-driven cancers.

## 2 Materials and methods

### 2.1 mRNA design, synthesis, and formulation

The CD80-E7 fusion protein was constructed by linking the extracellular and transmembrane domains of CD80 with HPV16 E7 (Uniprot P03129). The 4-1BBL-E6 fusion protein was constructed by linking the extracellular domain of 4-1BBL, the hinge and transmembrane domain of CD8a (Uniprot P01732), and HPV16 E6 (Uniprot P03126). The CD80-E7, 4-1BBL-E6, and full-length of CD70 were connected by P2A (GSGATNFSLLKQAGDVEENPGP) and T2A (GSGEGRGSLLTCGDVEENPGP) linkers to produce TriStim-E6/E7. For the murine TriStim-E6/E7 construct, mouse CD80 (Uniprot Q00609), 4-1BBL (Uniprot P41274), and CD70 (Uniprot O55237) sequences were used, while human CD80 (Uniprot P33681), 4-1BBL (Uniprot P41273), and CD70 (Uniprot P32970) sequences were used for the human TriStim-E6/E7 construct.

For the E6/E7-CD80/4-1BBL construct, the mouse CD70 and T2A linker were removed from the murine TriStim-E6/E7 construct. For the E6/E7-CD80/OX40L/CD70 construct, the extracellular domain of mouse 4-1BBL in the murine TriStim-E6/E7 construct was replaced with that of mouse OX40L (Uniprot P43488). For the E6/E7-CD80/4-1BBL/OX40L construct, mouse CD70 in the murine TriStim-E6/E7 construct was replaced with mouse OX40L.

For the E6-P2P16-MITD or E7-P2P16-MITD construct, HPV16 E6 or E7 sequences were inserted between the secretory signal peptide of human HLA and the P2P16 amino acid sequences from tetanus toxoid (TT) of *Clostridium tetani*, followed by the transmembrane and cytosolic domains of human HLA (19). For the E6/E7-mut construct, sequences of E6 and E7 from HPV16 were modified and linked by a cleavable linker (RGRKRRS) as described previously (20).

For the Ubiquitin-E6/E7 construct, G76V-mutated ubiquitin B (Uniprot P0CG47) containing a 25-amino acid C-terminal extension (VGKLGRQDPPVATMVSKGEELFTGV) was linked to HPV16 E6, followed by HPV16 E7 (21, 22). For the CD40L/TLR4/CD70 construct, mouse CD40L (Uniprot P27548), constitutively activated mouse TLR4 (Uniprot Q9QUK6), and mouse CD70 (Uniprot O55237) were linked by P2A and T2A linkers (23).

The membrane-bound anti-CD3ϵ antibody (mb-α-CD3) construct was engineered as follows: The single-chain variable fragment (scFv) of the anti-mouse CD3ϵ antibody (clone 145-2C11) (24) was generated by linking the amino acid sequences of the heavy chain and light chain variable domains using a flexible peptide linker (GSTSGSGKPGSGEGSTKG). The CD8α signal peptide (MASPLTRFLSLNLLLLGESIILGSGEA) was fused to the N-terminus of the anti-CD3ϵ scFv. This construct was further extended by incorporating the hinge and transmembrane domains of mouse CD8α (UniProt P01731), resulting in the membrane-bound anti-CD3ϵ antibody (mb-α-CD3).

The protein sequences were back-translated, and the corresponding DNA sequences were optimized for enhanced protein expression using a population immune algorithm, an in-silico analysis algorithm that leverages principles from both population genetics and immunology. The optimized open reading frames, flanked by 5’UTR, 3’UTR, and poly-A sequences, were subsequently integrated into plasmid vectors.

The HPV vaccine mRNA was produced and encapsulated in LNP using the Liverna Therapeutics platform (China patent ZL201911042634.2), as described previously (25–27). Briefly, the mRNA was synthesized through *in vitro* transcription and subsequently purified using an oligo-dT affinity column (Sepax Technologies, Inc., Newark, NJ, USA) followed by tangential flow filtration (Repligen Corporation, Waltham, MA, USA). The purified mRNA was then encapsulated in LNPs as described previously. Specifically, the ionizable lipid MC3, DSPC, cholesterol, and DMG-PEG2000, at a molar ratio of 49.1:6.9:41.1:2.9, were dissolved in ethanol to achieve a final concentration of 20 mM. The lipid mixture was rapidly mixed with a 10 mM citrate buffer (pH 4.0, with 130 mM NaCl) containing 0.2 mg/mL of mRNA at a ratio of 1:3 (lipid/mRNA, v/v), using a microfluidic device (Precision Nanosystems Inc., Vancouver, British Columbia, Canada). The product was analytically characterized by assessing the particle size, polydispersity, and encapsulation efficiency. The mRNA concentration in the final product was determined using a UV spectrometer (TU-1810PC, Persee Inc., Beijing, China).

### 2.2 Demonstration of TriStim-E6/E7 mRNA expression

To evaluate the *in vitro* expression of the TriStim-E6/E7, 5 μg of TriStim-E6/E7 mRNA was transfected into HEK293 cells (1 × 10^6^ cells, American Type Culture Collection, Manassas, VA, USA) using Lipofectamine^®^ MessengerMAX™ transfection reagent (Invitrogen, Carlsbad, CA, USA). Twenty-four hours after transfection, cells were collected for western blot analysis using anti-HPV16 E7 antibody (Cat. #ab308180, Abcam, Waltham, MA, USA) followed by HRP-conjugated anti-rabbit IgG antibody (Cat. #D-110058-0100, Sangon Biotech Inc., Shanghai, China). For the immunofluorescence assay, transfected HEK293 cells were fixed with 4% paraformaldehyde, permeabilized with 0.1% Triton X-100, and then blocked with 5% bovine serum albumin (BSA). Subsequently, the cells were stained with a fluorescein-conjugated anti-HPV16 E6 antibody (Cat. #sc-460, Santa Cruz, Dallas, TX, USA), followed by counterstaining with 4’,6-diamidino-2-phenylindole (DAPI; Cat. #C1005, Beyotime, Shanghai, China). Finally, the cells were mounted in anti-fluorescence quenching medium and observed under a fluorescence microscope. For assessing T cell co-stimulatory molecule expression, transfected HEK293 cells were stained with fluorescein-conjugated anti-mouse CD80 (Cat. #104705, BioLegend, San Diego, CA, USA), phycoerythrin-conjugated anti-mouse 4-1BBL (Cat. #107105, BioLegend, San Diego, CA, USA), and PerCP/Cyanine5.5-conjugated anti-mouse CD70 (Cat. #104613, BioLegend, San Diego, CA, USA) antibodies and then assessed using a CytoFLEX flow cytometer (Beckman Coulter, Indianapolis, IN, USA), and the flow cytometry data were processed using FlowJo software v10 (Tree Star Inc., Ashland, OR, USA).

### 2.3 Immunization and analysis of cellular immune response

C57BL/6 female mice (6–8 weeks old, 18–23 g) from Zhuhai BesTest BioTech Co., Ltd. were randomly divided into 8 groups and intramuscularly administered with one, two, or three doses of TriStim-E6/E7 mRNA (25 μg/mouse/injection) or control empty LNPs (100 μL/mouse/injection). The 0d group was immunized on day 0 and sacrificed on day 14. The 0d/7d group was immunized on days 0 and 7, then sacrificed on day 21. The 0d/14d group was immunized on days 0 and 14, then sacrificed on day 28. The 0d/7d/14d group was immunized on days 0, 7, and 14, then sacrificed on day 28.

For human TriStim-E6/E7 mRNA immunization, CD28/4-1BB/CD27 triple-humanized mice from Shanghai Model Organisms Center, Inc. were used. Mice intramuscularly received 20 μg of human TriStim-E6/E7 mRNA or 100 μL of empty LNPs on days 0, 7, and 14, were sacrificed on day 28.

Peripheral blood from immunized mice were collected, stained with phycoerythrin-conjugated H2-D^b^ HPV16 E7 Tetramer-RAHYNIVTF (Cat. #TB-5008-1, Medical & Biological Laboratories Co., Ltd, Tokyo, Japan), or phycoerythrin-conjugated H2-K^b^ HPV16 E6 Tetramer-EVYDFAFRDL (Cat. #JD03600, Immudex, Brede, Denmark), and analyzed by flow cytometry. To assess antigen-specific cytokine production, splenocytes were stimulated with 5 μg/ml of HPV16 E6 and E7 peptide library mixture (GenScript, Nanjing, China) for 48 h. Brefeldin A (Cat. #420601, BioLegend) was added 18 h before harvesting to inhibit intracellular cytokine secretion. Cells were then collected and subjected to cell surface staining with fluorescein-conjugated anti-mouse CD3 (Cat. #100204, BioLegend), Brilliant Violet 510-conjugated anti-mouse CD4 (Cat. #100559, BioLegend) and Brilliant Violet 605-conjugated anti-mouse CD8 (Cat. #100744, BioLegend) antibodies, followed by fixation and permeabilization using Cytofix/Cytoperm™ Kit (Cat. #554714, BD Biosciences, Franklin Lakes, NJ, USA) and intracellular staining with Brilliant Violet 421-conjugated anti-mouse IFN-γ (Cat. #100767, BioLegend) and phycoerythrin-conjugated anti-mouse TNF-α (Cat. #104789, BioLegend) antibodies. The cells were then analyzed using flow cytometry according to the manufacturer’s instructions.

To evaluate *in vitro* antigen-specific cell lysis, splenocytes from immunized mice were stimulated with 5 μg/ml of HPV16 E6 and E7 peptide library mixture for 48 h, then co-cultured with TC-1/Luc cells (stably expressing luciferase gene) at an effector-to-target cell ratio of 10:1. After 24 h, cells were collected and analyzed for luciferase activity using a luciferase reporter gene assay kit (Cat. #11401ES76, Yeasen, Shanghai, China) according to the manufacturer’s instructions.

### 2.4 *In vivo* anti-tumor efficacy analysis

Syngeneic HPV^+^ tumor models were established in C57BL/6 mice via subcutaneous injection of HPV16^+^ TC-1 cells (1.5 × 10^5^ in 100 μL DPBS) in the right flank. For human TriStim-E6/E7 mRNA immunization, CD28/4-1BB/CD27 triple-humanized mice from Shanghai Model Organisms Center, Inc. were used. Tumor width and length were measured 2–3 times weekly with calipers, and volume was calculated as (length × width²)/2. When average tumor volume reached approximately 80–100 mm³ (typically at 5–6 days after tumor injection), mice were randomly grouped for treatment. LNP-encapsulated mRNA was intramuscularly injected weekly at the indicated dose for 3 consecutive weeks, and empty LNPs was used as controls. For antibody treatment, anti-mouse PD-L1 (BioXCell, Cat. #BE0101), anti-mouse CD4 (BioXCell, Cat. #BE0119), anti-mouse CD8 antibodies (BioXCell, Cat. #BE0061) or isotype control (BioXCell, Cat. #BE0090) were intraperitoneally injected at 200 μg/mouse/injection, twice weekly for 3 consecutive weeks as indicated in figure legends. In combination treatment group, both mRNA and antibody were first administered on day 0. Mice were monitored daily for adverse reactions, with tumor size and body weight measured 2–3 times weekly. Survival was recorded when mice died or were euthanized due to excessive tumor growth (defined as >3000 mm³ for individual mice and >2000 mm³ for the group average) or severe adverse effects. To evaluate E7−specific cellular immunity, peripheral blood was collected at the indicated day. Red blood cells were lysed, and the remaining cells were stained with phycoerythrin-conjugated H2-D^b^ HPV16 E7 Tetramer-RAHYNIVTF (Cat. #TB-5008-1, Medical & Biological Laboratories Co., Ltd, Tokyo, Japan), fluorescein-conjugated anti-mouse CD3 (Cat. #100204, BioLegend), Brilliant Violet 510-conjugated anti-mouse CD4 (Cat. #100559, BioLegend) and Brilliant Violet 605-conjugated anti-mouse CD8 (Cat. #100744, BioLegend) antibodies, followed by flow cytometry analysis.

For the rechallenge experiments, TC-1 tumor-bearing mice were intramuscularly injected with 25 μg/mouse/injection of TriStim-E6/E7 mRNA weekly for 3 consecutive weeks. Mice that exhibited complete tumor regression were rechallenged with autologous TC-1 tumor cells at distal sites 40 days after the initial tumor challenge. Naive mice were challenged with the same tumor as negative controls. Mice were monitored daily for adverse reactions, and tumor size and body weight were measured 2–3 times per week.

For the tumor prevention experiment, C57BL/6 mice were intramuscularly injected with either 5 μg or 25 μg of TriStim-E6/E7 mRNA per injection weekly for 3 consecutive weeks. Following the vaccination regimen, mice underwent 3 tumor challenges with TC-1 cells at 21, 56, and 125 days after the first vaccination, respectively. Peripheral blood was collected before and after the third tumor challenge (i.e., at 124 and 131 days post-first vaccination) and analyzed by E7 tetramer staining as described above. Naive mice were challenged with the same tumor on the same day as negative controls.

### 2.5 Analysis of tumor infiltrating lymphocytes

C57BL/6 mice were intramuscularly injected with 3 μg of TriStim-E6/E7 mRNA or control empty LNPs once a week for 3 consecutive weeks. Mice were then subcutaneously challenged with TC-1 tumor cells (1.5 × 10^5^ in 100 μL DPBS), 21 days after the first vaccination. Tumors were resected 2 days later for TILs analysis. Tumor samples were cut into small pieces and incubated in RPMI 1640 medium (Cat. #11875093, Thermo Fisher, Waltham, MA, USA) containing 1 mg/ml of collagenase IV (Cat. #40510ES60, Yeasen, Shanghai, China), 0.2 mg/ml of hyaluronidase (Cat. #20426ES60, Yeasen, Shanghai, China), and 0.2 mg/ml of deoxyribonuclease I (Cat. #10608ES60, Yeasen, Shanghai, China), for 60 min with shaking. The cell suspensions were filtered through 40-μm cell strainers (Cat. #84701ES50, Yeasen, Shanghai, China) and washed with RPMI 1640 medium containing 10% FBS. Cells were then stained with phycoerythrin-conjugated H-2Db HPV16 E7 Tetramer-RAHYNIVTF (Cat. #TB-5008-1, Medical & Biological Laboratories Co., Ltd, Tokyo, Japan), fluorescein-conjugated anti-mouse CD3 (Cat. #100204, BioLegend), Brilliant Violet 510-conjugated anti-mouse CD4 (Cat. #100559, BioLegend) and Brilliant Violet 605-conjugated anti-mouse CD8 (Cat. #100744, BioLegend) antibodies, followed by flow cytometry analysis.

### 2.6 *In vitro* functional analysis

To evaluate the *in vitro* function of TriStim-E6/E7, HEK293 cells (1 × 10^6^) were co-transfected with 0.25 μg of mb-α-CD3 mRNA and 5 μg of TriStim-E6/E7 mRNA. Twenty-four hours post-transfection, these cells were used to stimulate mouse splenocytes. Control groups included cells transfected with empty LNPs, 0.25 μg of mb-α-CD3 mRNA alone, or combination of 0.25 μg of mb-α-CD3 mRNA and 5 μg of E6/E7-P2P16-MITD mRNA. After 48 hours of co-culture, the cells were harvested and surface-stained with fluorescein-conjugated anti-mouse CD3 (Clone 145-2C11, Cat. #100204, BioLegend), PE-conjugated anti-mouse CD25 (Cat. #113704, BioLegend), and BD Horizon BB700-conjugated anti-mouse CD69 (Cat. #566500, BD Biosciences, Franklin Lakes, NJ, USA). Flow cytometric analysis was performed according to the manufacturer’s instructions.

To assess T cell proliferation, mRNA-transfected HEK293 cells were treated with 15 μg/mL mitomycin C (Cat. #HY-13316, MedChemExpress, Shanghai, China) for 4 hours to inhibit cell growth. After two washes to remove mitomycin, the HEK293 cells were used to stimulate mouse splenocytes that had been pre-stained with carboxyfluorescein succinimidyl ester (CFSE) (Cat. #423801, BioLegend). After 72 hours of co-culture, the cells were harvested, surface-stained with Brilliant Violet 510-conjugated anti-mouse CD4 (Cat. #100559, BioLegend) and Brilliant Violet 605-conjugated anti-mouse CD8 (Cat. #100744, BioLegend) antibodies and analyzed for T cell proliferation using flow cytometry.

### 2.7 Quantification of human cytokine production in PBMC-NSG mice

A humanized immune system mouse model was established by intravenous injection of human PBMCs (1×10^7^ cells in 200 μL DPBS per mouse) into M-NSG mice (purchased from Shanghai Model Organisms Center, Inc.). Twenty days post-PBMC engraftment, the reconstituted human leukocytes were confirmed by flow cytometry analysis of mouse peripheral blood samples using anti-human CD45 (Cat. #563879, BD-Biosciences, San Jose, CA) antibody. Twenty-one days post-PBMC engraftment, mice were intramuscularly injected with 20 μg of human TriStim-E6/E7 mRNA, while empty LNPs were administered as a negative control. Intravenous administration of TGN1412 (Cat. #286306, MedChemExpress, Shanghai, China) at a dose of 2 mg/kg served as a positive control. Mice were bled at 6 h and 24 h post-injection, and serum was collected and analyzed for human cytokines (IFNγ, IL-10, IL-6, IL-2, IL-4, and TNFα) using a LEGENDplex™ Human Cytokine Kit (Cat. #741195, 740270, 741191, 740268, 740269 and 741192, BioLegend).

### 2.8 Statistical analysis

Statistical analyses were performed using GraphPad Prism 8 software (La Jolla, CA, USA). Data were presented as the mean ± standard error of the mean or the mean ± standard deviation, as specified. Multiple groups were compared using unpaired two tailed Student’s t-tests, two-way ANOVA with Sidak’s multiple comparison test, or Log-rank (Mantel-Cox) tests, as indicated in the figure legends. An adjusted P value of ≤ 0.05 was considered indicative of a statistically significant difference. Statistical differences are denoted as *P ≤ 0.05, **P ≤ 0.01, and ***P ≤ 0.001.

## 3 Results

### 3.1 Development of a novel HPV mRNA vaccine, encoding E6/E7 and T cell co-stimulatory molecules

**Figure 1:**
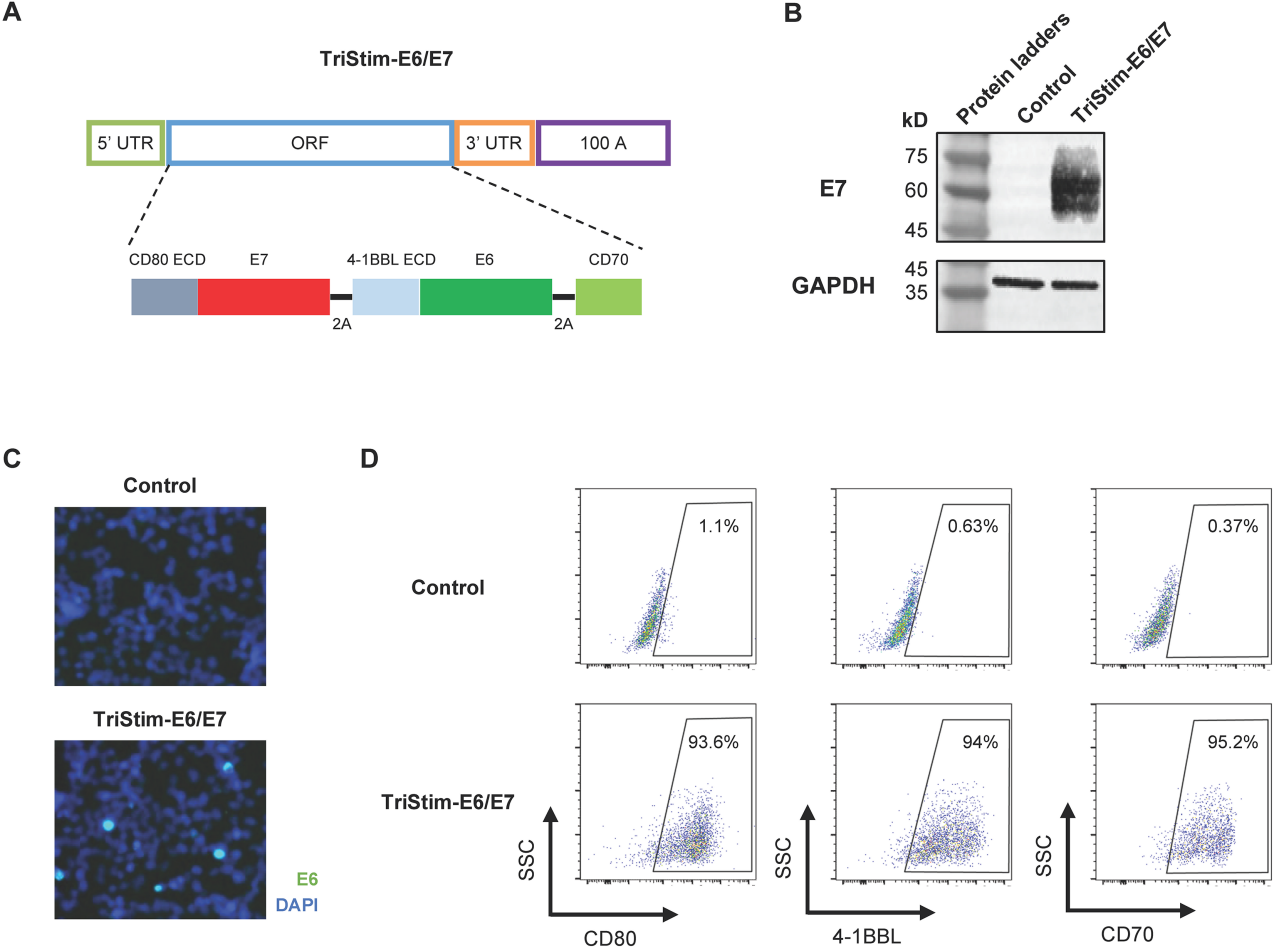
TriStim-E6/E7 mRNA expressed *in vitro*. **(A)** Schematic diagram of the TriStim-E6/E7 mRNA construct. **(B)** The expression of CD80-E7 fusion protein (~60 kDa) was detected in mRNA-transfected HEK293 cells by western blotting. **(C)** The expression of E6 was detected in mRNA-transfected HEK293 cells by immunofluorescence microscopy. **(D)** The expression of CD80, 4-1BBL and CD70 was detected in mRNA-transfected HEK293 cells by flow cytometry. Data are representative of three independent experiments.

The E6 and E7 oncoproteins of human papillomavirus 16 (HPV16) represent well-established therapeutic targets for HPV-associated malignancies (6). While CD80, 4-1BBL, and CD70 are critical T-cell co-stimulatory molecules governing antigen-specific T-cell activation, expansion, and long-term persistence (13), their limited interspecies homology (<60% sequence similarity between human and murine counterparts) poses challenges for preclinical evaluation in murine models. To address this species-specificity barrier while simultaneously enhancing antigen presentation and immune activation, we developed TriStim-E6/E7, an HPV mRNA vaccine encoding three components linked by cleavable 2A linkers: a fusion protein of the extracellular domain of murine CD80 and HPV16 E7, a fusion protein of the extracellular domain of murine 4-1BBL and HPV16 E6, and full-length murine CD70 (**Figure 1A**). TriStim-E6/E7 mRNA was transfected into HEK293 cells. Successful expression of E7 was confirmed via western blot (**Figure 1B**), while the expression of E6 was validated by immunofluorescence microscopy (**Figure 1C**). Furthermore, surface expression of CD80, 4-1BBL, and CD70 was verified by flow cytometry (**Figure 1D**).

### 3.2 TriStim-E6/E7 mRNA induced a robust antigen-specific cellular immune response

**Figure 2:**
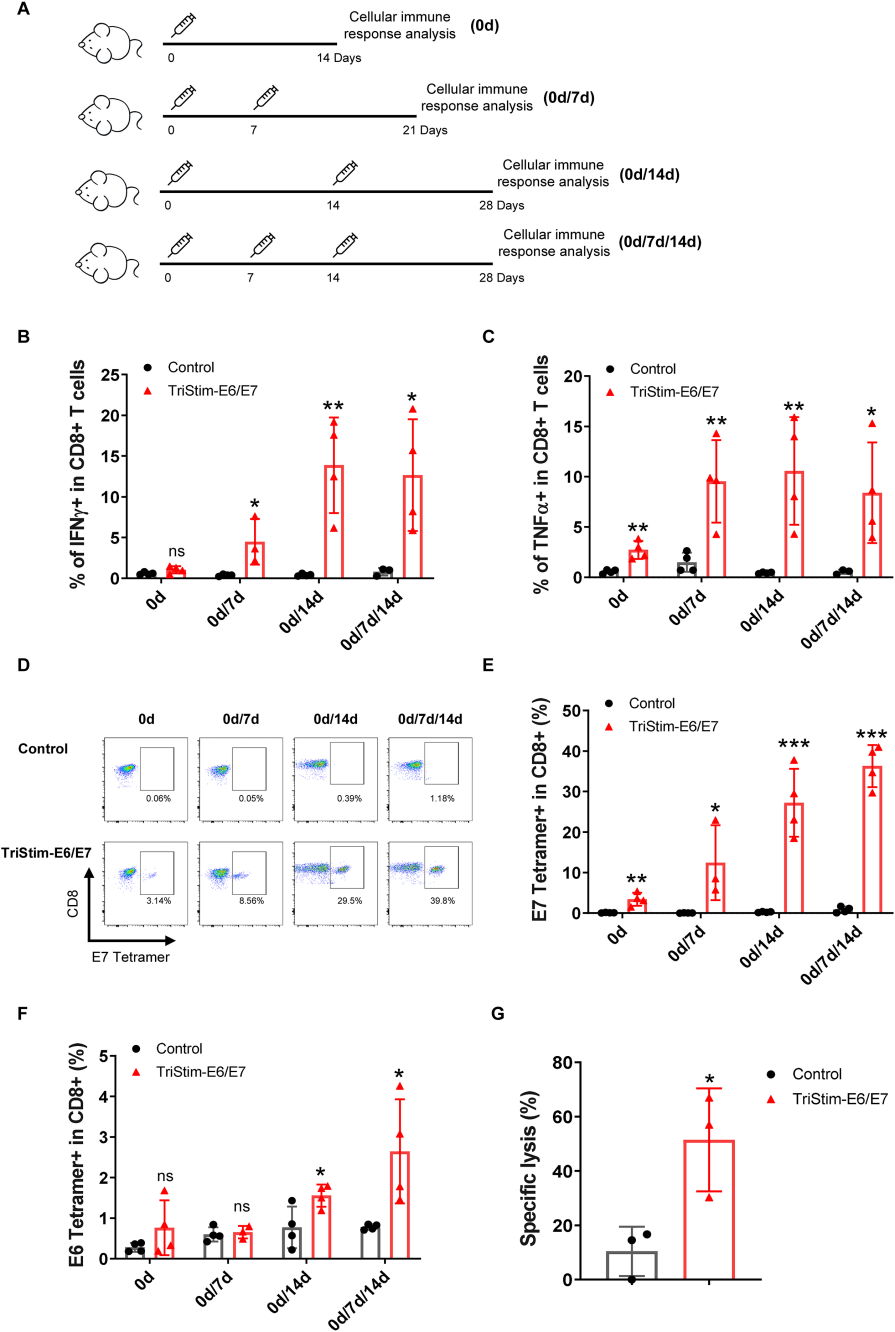
TriStim-E6/E7 mRNA induced a robust antigen-specific cellular immune response in mice. **(A)** Scheme of vaccination. Mice were intramuscularly vaccinated with one, two, or three doses of 25 μg TriStim-E6/E7 mRNA-LNP or control empty LNPs, and the cellular immune responses were assessed. **(B, C)** IFNγ-producing CD8^+^ T cells and TNFα-producing CD8^+^ T cells were measured using flow cytometry in splenocytes stimulated with HPV16 E6 and E7 peptide pools. **(D-F)** E7 tetramer^+^ CD8^+^ T cells and E6 tetramer^+^ CD8^+^ T cells in peripheral blood of immunized mice were measured using flow cytometry. Representative flow cytometry plots of E7 tetramer^+^ CD8^+^ T cells **(D)** and quantification of E7 tetramer^+^ CD8^+^ T cells **(E)** and E6 tetramer^+^ CD8^+^ T cells **(F)** are presented. **(G)** Splenic T cells from vaccinated mice induced cytotoxicity against TC-1 tumor cells. Data are presented as the mean ± standard deviation, with n = 4 **(B, C, E, F)** and n = 3 **(G)**. Statistical analysis was conducted using an unpaired, two-tailed Student’s t-test; ns, not significant, *P ≤ 0.05, **P ≤ 0.01, ***P ≤ 0.001.

Antigen-specific cellular immune responses are critical for the anti-tumor efficacy of tumor vaccines. In this study, mice were intramuscularly vaccinated with one (day 0), two (days 0 and 7 or days 0 and 14), or three doses (days 0, 7, and 14) of 25 μg TriStim-E6/E7 mRNA, followed by assessment of cellular immune responses (**Figure 2A**). Vaccination with two or three doses significantly increased the number of IFN-γ- and TNF-α-producing cells compared to control empty LNPs vaccination (**Figures 2B, C**). Among the tested groups, the two-dose regimen with a 14-day interval induced the highest cytokine production.

The well-characterized murine H2-D^b^ restricted CD8^+^ epitopes E7_49–57_ and E6_48–57_ were used to evaluate antigen-specific T cell responses (28–30). TriStim-E6/E7 vaccination markedly increased the percentage of E7 tetramer (E7_49-57_/H2-D^b^ restricted)^+^ CD8^+^ T cells, reaching 36.2% after three inoculations (**Figures 2D, E**). In contrast, E6 tetramer (E6_48-57_/H2-D^b^ restricted)^+^ CD8^+^ T cells were barely detectable, with only a slight increase after three inoculations (**Figure 2F**). Furthermore, CD8^+^ T cells derived from splenocytes of vaccinated mice exhibited cytotoxicity against HPV-positive TC-1 tumor cells *in vitro* (**Figure 2G**). Collectively, these findings demonstrate that TriStim-E6/E7 mRNA induces a robust antigen-specific cellular immune response, particularly targeting the E7 antigen.

### 3.3 TriStim-E6/E7 mRNA induced tumor regression in a syngeneic tumor model

**Figure 3:**
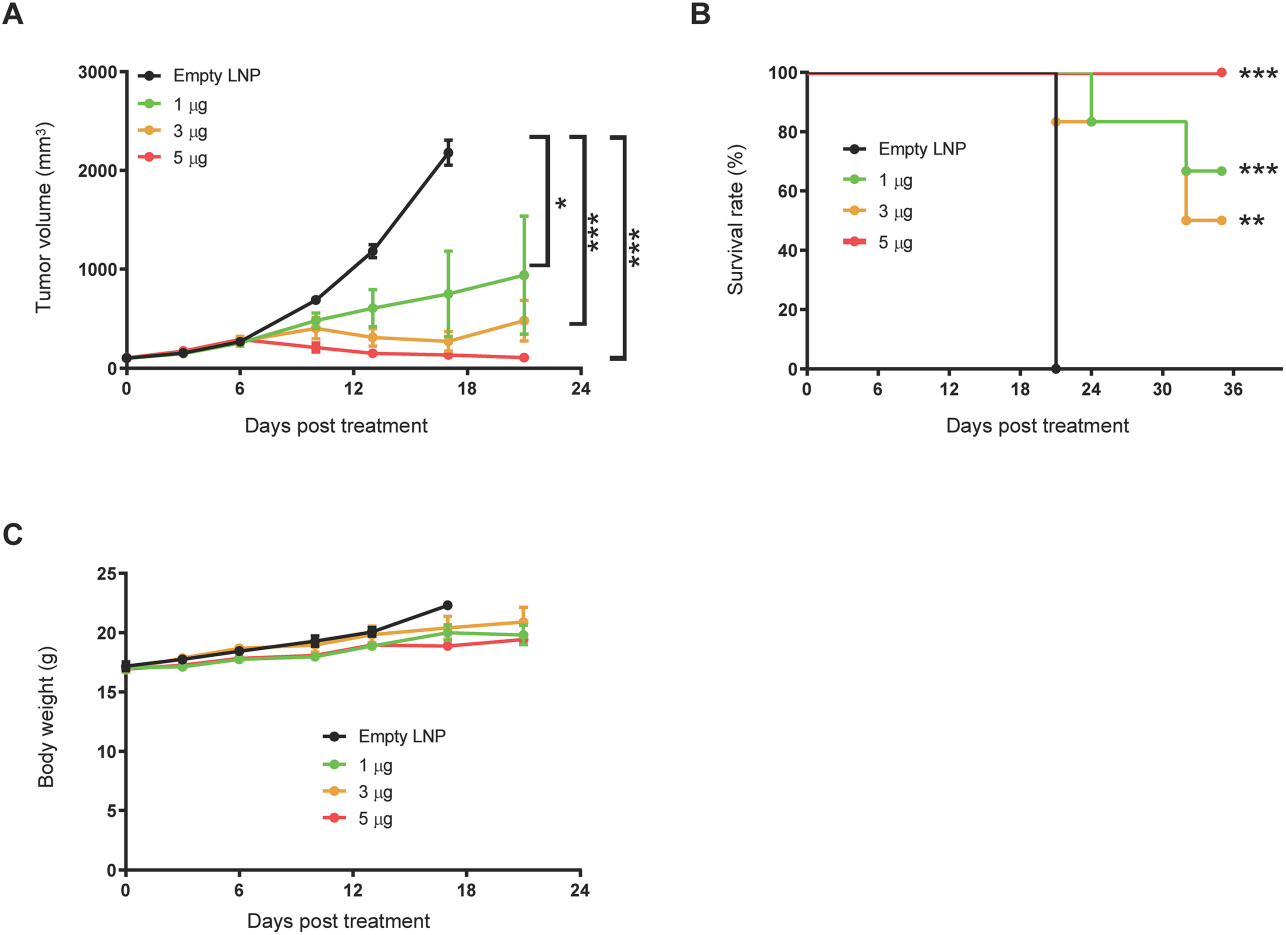
TriStim-E6/E7 mRNA inhibited tumor growth in a syngeneic TC-1 tumor-bearing mouse model. Mice bearing subcutaneous TC-1 tumors were treated with 1, 3 or 5 μg of TriStim-E6/E7 mRNA once a week for 3 consecutive weeks (on day 0, 7 and 14 after grouping), and their tumor volumes **(A)** and body weights **(C)** were measured, and Kaplan–Meier survival curves **(B)** were generated. Data are presented as the mean ± standard error of the mean (n = 6). Statistical analysis for tumor volumes was conducted with two-way ANOVA; *P ≤ 0.05, ***P ≤ 0.001. Survival curves were analyzed with a Log-rank (Mantel-Cox) test; **P ≤ 0.01, ***P ≤ 0.001.

To evaluate the therapeutic efficacy of TriStim-E6/E7, an HPV16^+^ tumor model was established by subcutaneous inoculation of TC-1 cells, which express both E6 and E7 oncogenes. Mice were treated with repeated intramuscular administrations of TriStim-E6/E7 mRNA. While 1 μg of TriStim-E6/E7 mRNA showed moderate efficacy in restricting tumor growth, treatment with 5 μg and 25 μg doses resulted in significant tumor growth inhibition, with tumor growth inhibition index (TGI) of 88.9% and 96.3%, respectively, 21 days after the first treatment (**Figure 3A**). Furthermore, TriStim-E6/E7 mRNA treatment significantly prolonged the survival of tumor bearing mice (**Figure 3B**). TriStim-E6/E7 mRNA treatment was well tolerated, with no significant body weight loss (**Figure 3C**) or obvious adverse reactions observed during the experimental period.

**Figure 4:**
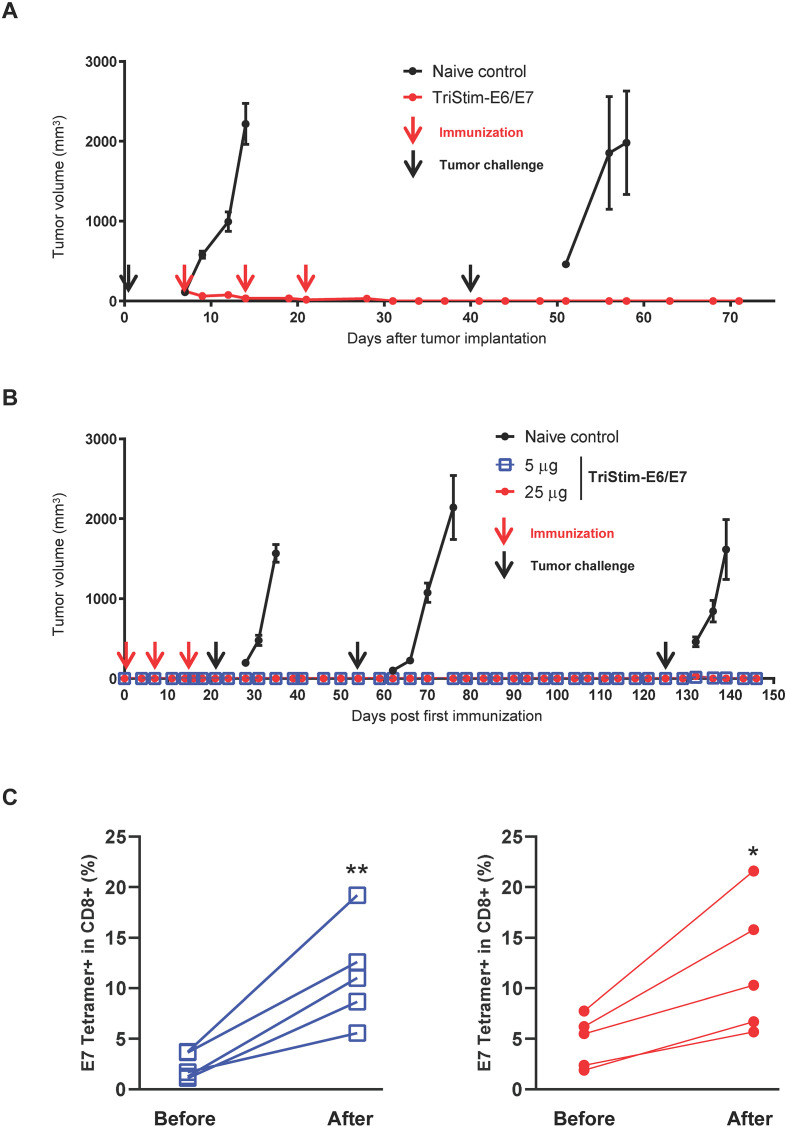
TriStim-E6/E7 mRNA induced anti-tumor immune memory. **(A)** C57BL/6 mice were subcutaneously implanted with TC-1 tumor and followed by the treatment of 25 μg of TriStim-E6/E7 mRNA once a week for 3 consecutive weeks. Forty days after tumor implantation, mice cured by TriStim-E6/E7 mRNA vaccination were rechallenged with an autologous TC-1 tumor, and their tumor volume was determined. Naïve mice were challenged with the same tumor at the same time as controls. **(B)** C57BL/6 mice were intramuscularly vaccinated with 5 or 25 μg of TriStim-E6/E7 mRNA once a week for 3 consecutive weeks. Mice were then challenged three times with TC-1 cells at 21, 56, and 125 days after the first vaccination, respectively. Their tumor volume was determined. Naïve mice were challenged with the same tumor at the same time as controls. **(C)** Peripheral blood of mice in **(B)** was collected before and after the third tumor challenge (i.e., at 124 and 131 days post-first vaccination) and analyzed for E7 tetramer^+^ CD8^+^ cells by flow cytometry. Blue lines (left panel) indicate data generated from mice vaccinated with 5 μg of TriStim-E6/E7 mRNA, while the red lines (right panel) indicate data generated from mice vaccinated with 25 μg of TriStim-E6/E7 mRNA. Data **(A, B)** are presented as the mean ± standard error of the mean (n = 6). Statistical analysis was conducted using an unpaired, two-tailed Student’s t-test; *P ≤ 0.05, **P ≤ 0.01.

Mice cured by TriStim-E6/E7 mRNA vaccination demonstrated resistance to rechallenge with autologous TC-1 tumors at distal sites, while all naïve mice developed significant tumor progression (**Figure 4A**). This indicates the development of anti-tumor immunological memory in vaccinated mice.

To assess the duration of anti-tumor immune memory, mice receiving three doses of TriStim-E6/E7 mRNA were challenged with TC-1 tumors three times at days 21, 56, and 125 post-first vaccination. No vaccinated mice developed tumor lesions upon challenge, while all naïve mice succumbed to tumor progression (**Figure 4B**). Furthermore, a medium level of E7-specific T cells was detected in the peripheral blood of mice 124 days after the first vaccination and before the third tumor challenge, with significant elevation upon the third challenge (**Figure 4C**).

These results collectively demonstrate that TriStim-E6/E7 mRNA induces long-lasting antigen-specific immune memory and provides sustained protection against tumors expressing the target antigens.

### 3.4 TriStim-E6/E7 mRNA induced superior anti-tumor potency

Various approaches have been employed to enhance cellular immune responses through improving antigen processing and presentation and inducing T cell activation. We synthesized mRNAs based on the information from literature and evaluated their *in vivo* anti-tumor activity compared to TriStim-E6/E7.

**Figure 5:**
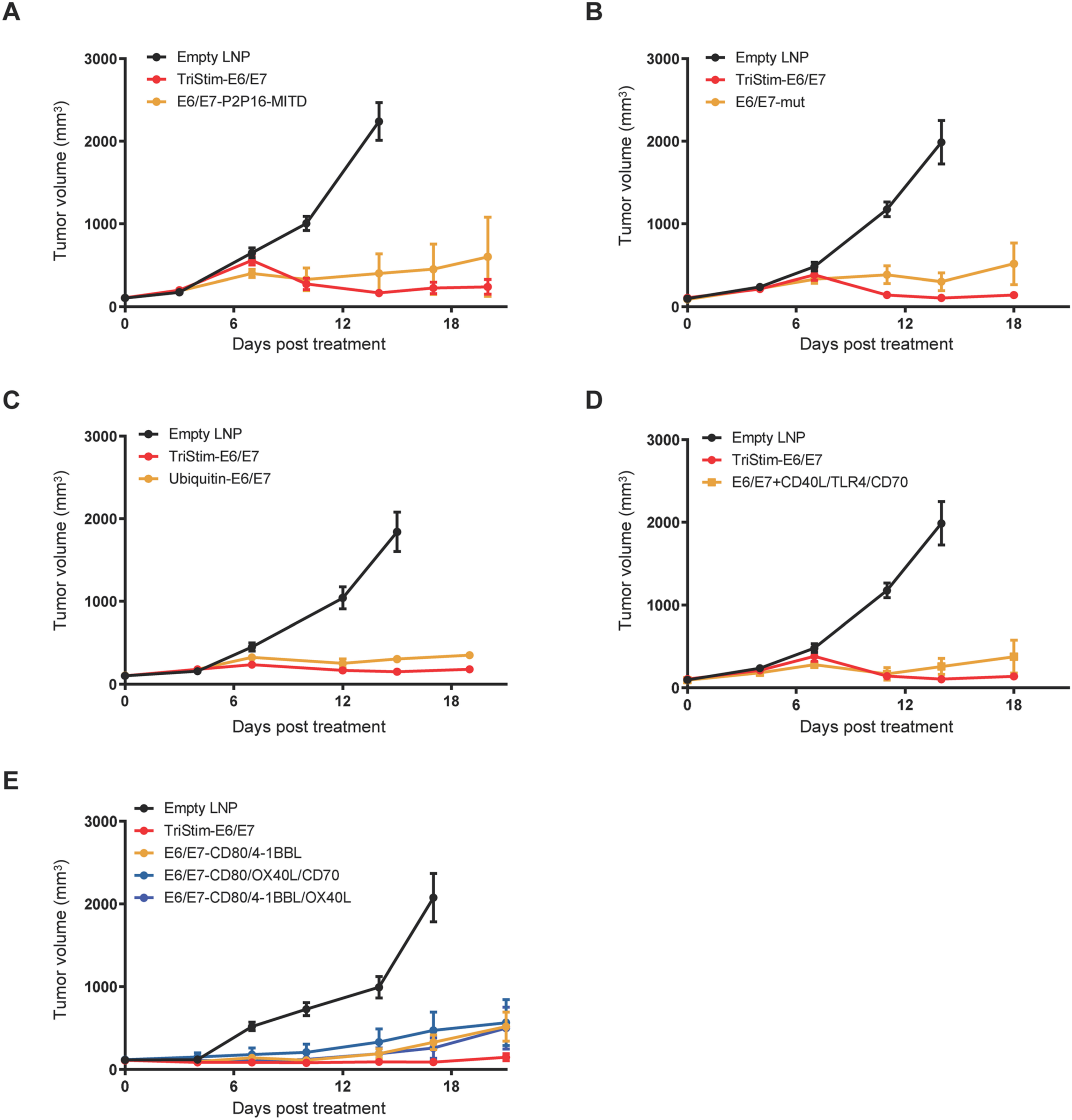
TriStim-E6/E7 mRNA induced superior anti-tumor potency. Mice bearing subcutaneous TC-1 tumors were treated with 5 μg of TriStim-E6/E7 mRNA once a week for 3 consecutive weeks (on day 0, 7 and 14 after grouping), and their tumor volumes were measured. Treatment with the same dose of E6/E7-P2P16-MITD mRNA **(A)**, E6/E7-mut mRNA **(B)**, Ubiquitin-E6/E7 mRNA **(C)**, combination of E6/E7-mut mRNA and CD40L/TLR4/CD70 mRNA **(D)**, E6/E7-CD80/4-1BBL mRNA **(E)**, E6/E7-CD80/OX40L/CD70 mRNA **(E)**, E6/E7-CD80/4-1BBL/OX40L mRNA **(E)** was used for comparison. Data are presented as the mean ± standard error of the mean (n = 6).

One approach involved fusing the antigen with P2P16 amino acid sequences from tetanus toxoid (TT) of *Clostridium tetani*, potentially overcoming immunological self-tolerance by providing tumor-unspecific T cell help during priming. This was further enhanced by fusion with the transmembrane and cytoplasmic domain of MHC class I (MITD), improving antigen processing and presentation (19). The combination of E6-P2P16-MITD mRNA and E7-P2P16-MITD mRNA achieved 86.7% tumor growth inhibition (TGI) 20 days post-first dose, slightly lower than TriStim-E6/E7 mRNA’s 94.7% TGI (**Figure 5A**).

Other construct E6/E7-mut mRNA (encoding mutated E6 and E7 from HPV16) (20), resulted in 87.6% TGI, 18 days post first dose. In this experiment, TriStim-E6/E7 mRNA demonstrated higher efficacy, achieving 96.8% TGI compared to these constructs (**Figure 5B**).

Ubiqutin-E6/E7 mRNA, encoding a fusion of HPV16 E6/E7 with ubiquitin B to enhance antigen processing (21, 22), exhibited 87.5% TGI 19 days post-first dose, while TriStim-E6/E7 mRNA achieved 94.5% TGI (**Figure 5C**). Combination of CD40L/TLR4/CD70 mRNA (encoding CD40L, constitutively activated TLR4, and CD70, to enhance dendritic cell-mediated T cell stimulation) (23) and E6/E7 mRNA (encoding mutated E6 and E7 from HPV16) (20), resulted in 90.4% TGI 18 days post-first dose, compared to TriStim-E6/E7 mRNA’s 96.8% TGI (**Figure 5D**).

To investigate the role of T cell co-stimulatory molecules in TriStim-E6/E7 mRNA, we removed CD70 or replaced 4-1BBL or CD70 with OX40L. Combinations of antigens with CD80 and 4-1BBL achieved 75.4% TGI 17 days post-first dose. Replacing one co-stimulatory molecule with OX40L (either CD80/OX40L/CD70 or CD80/4-1BBL/OX40L) resulted in 78.1% and 79.6% TGI, respectively. In contrast, TriStim-E6/E7 mRNA achieved 86.4% TGI (**Figure 5E**).

Collectively, these data demonstrate the superior anti-tumor activity of TriStim-E6/E7 mRNA. The combination of CD80, 4-1BBL, and CD70 in TriStim-E6/E7 mRNA represents an optimal configuration for T cell co-stimulation under the current experimental conditions.

### 3.5 TriStim-E6/E7 mRNA induced anti-tumor activity was mediated by antigen-specific CD8^+^ T cells

**Figure 6:**
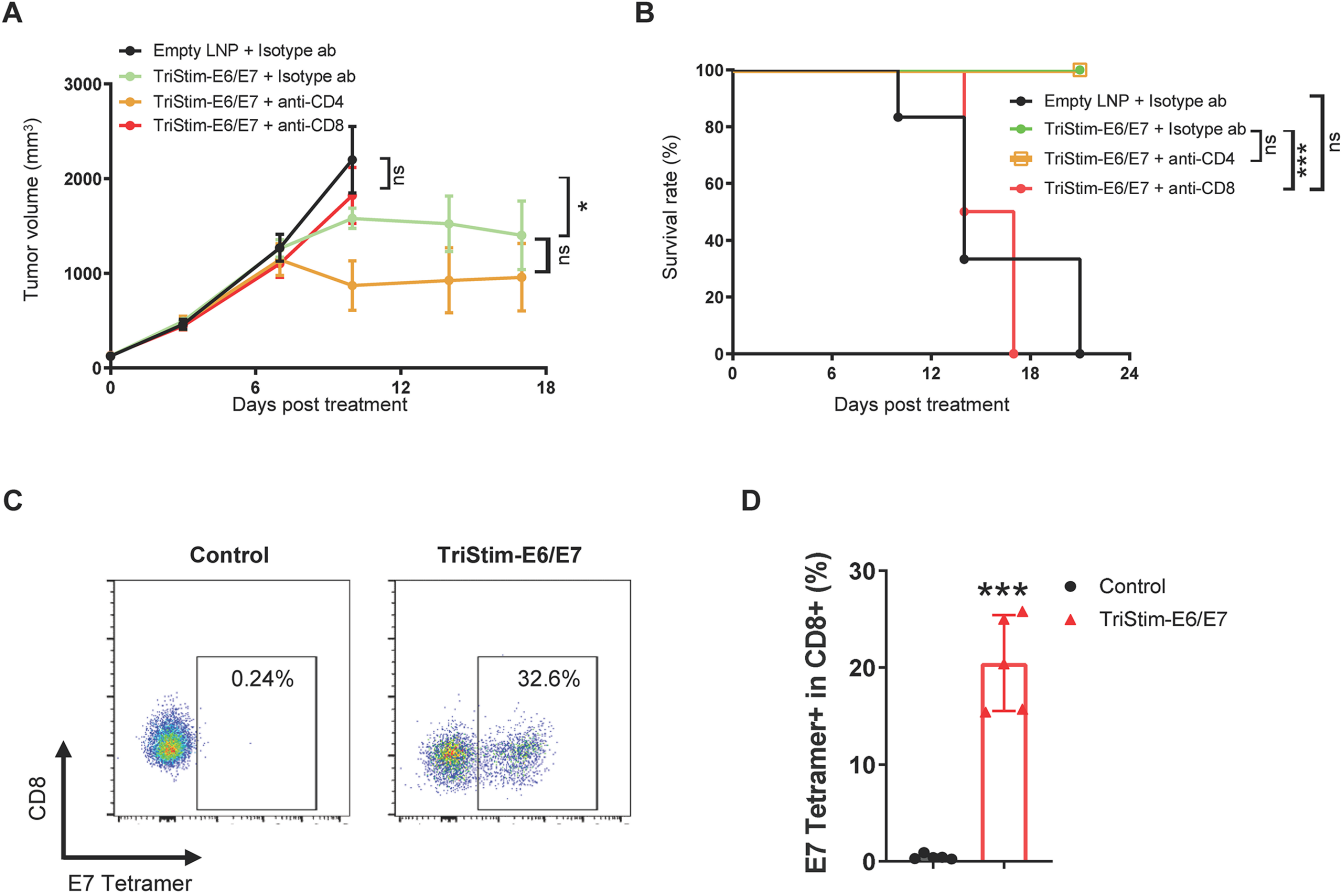
TriStim-E6/E7 mRNA induced anti-tumor activity was mediated by antigen-specific CD8^+^ T cells. **(A, B)** Mice bearing subcutaneous TC-1 tumors were treated with 5 μg of TriStim-E6/E7 mRNA once a week for 3 consecutive weeks (on day 0, 7 and 14 after grouping), and 200 μg of anti-CD4 or anti-CD8 monoclonal antibodies twice a week for 3 consecutive weeks (on day 0, 3, 7, 10 and 14 after grouping), and their tumor volumes **(A)** were measured, and Kaplan–Meier survival curves **(B)** were generated. **(C, D)** Mice immunized with 3 μg of TriStim-E6/E7 mRNA once a week for 3 consecutive weeks, were subcutaneously implanted with TC-1 tumor 21 days after the first immunization. Tumors were resected 2 days later for TILs analysis. Representative flow cytometry plots of E7 tetramer^+^ CD8^+^ T cells **(C)** and quantification of E7 tetramer^+^ CD8^+^ T cells **(D)** were presented. Data in **(A)** are presented as the mean ± standard error of the mean (n = 6). Data in **(D)** are presented as the mean ± standard deviation (n = 5). Statistical analysis for tumor volumes **(A)** was conducted through two-way ANOVA with Sidak’s multiple comparison test; ns, not significant, *P ≤ 0.05. Survival curves **(B)** were analyzed with a Log-rank (Mantel-Cox) test; ns, not significant, ***P ≤ 0.001. Statistical analysis for E7 tetramer^+^ CD8^+^ T cells **(D)** was conducted using an unpaired, two-tailed Student’s t-test; ***P ≤ 0.001.

Both CD4^+^ and CD8^+^ T cells play crucial roles in immune responses against pathogens (31). To investigate the contribution of these T cell subtypes to the immune response elicited by TriStim-E6/E7 mRNA and its anti-tumor activity, we depleted CD4^+^ and CD8^+^ T cells using neutralizing antibodies. Immunization with TriStim-E6/E7 mRNA significantly inhibited the growth of TC-1 tumors. This anti-tumor effect remained intact in the presence of CD4-neutralizing antibodies (**Figures 6A, B**). However, the addition of CD8-neutralizing antibodies completely abrogated the therapeutic efficacy of TriStim-E6/E7 mRNA, resulting in progressive tumor growth (**Figure 6A**) and reduced animal survival rate (**Figure 6B**) comparable to that observed in mice treated with empty LNPs.

In order to analyze tumor-infiltrating lymphocytes, mice immunized with 3 doses of TriStim-E6/E7 mRNA were subcutaneously implanted with TC-1 tumors, followed by tumor resection and flow cytometry analysis. E7 tetramer (E7_49-57_/H2-D^b^ restricted)^+^ CD8^+^ T cells were merely detected in control empty LNPs-treated mice but were significantly elevated upon TriStim-E6/E7 mRNA immunization, accounting for more than 20% of tumor-infiltrating CD8^+^ T cells (**Figures 6C, D**). Collectively, these data suggested that TriStim-E6/E7 mRNA induced anti-tumor activity was mediated by antigen-specific CD8^+^ T cells.

### 3.6 TriStim-E6/E7 mRNA induced T cell activation and proliferation *in vitro*

TriStim-E6/E7 mRNA encodes both E6/E7 antigens and T cell co-stimulatory molecules (CD80, 4-1BBL, and CD70). To verify the functionality of these co-stimulatory molecules, we conducted *in vitro* assays analyzing T cell activation and proliferation using HEK293 cells expressing membrane-bound anti-CD3ϵ antibody (mb-α-CD3) as T cell signal 1 and expressing T cell co-stimulatory molecules as signal 2.

**Figure 7:**
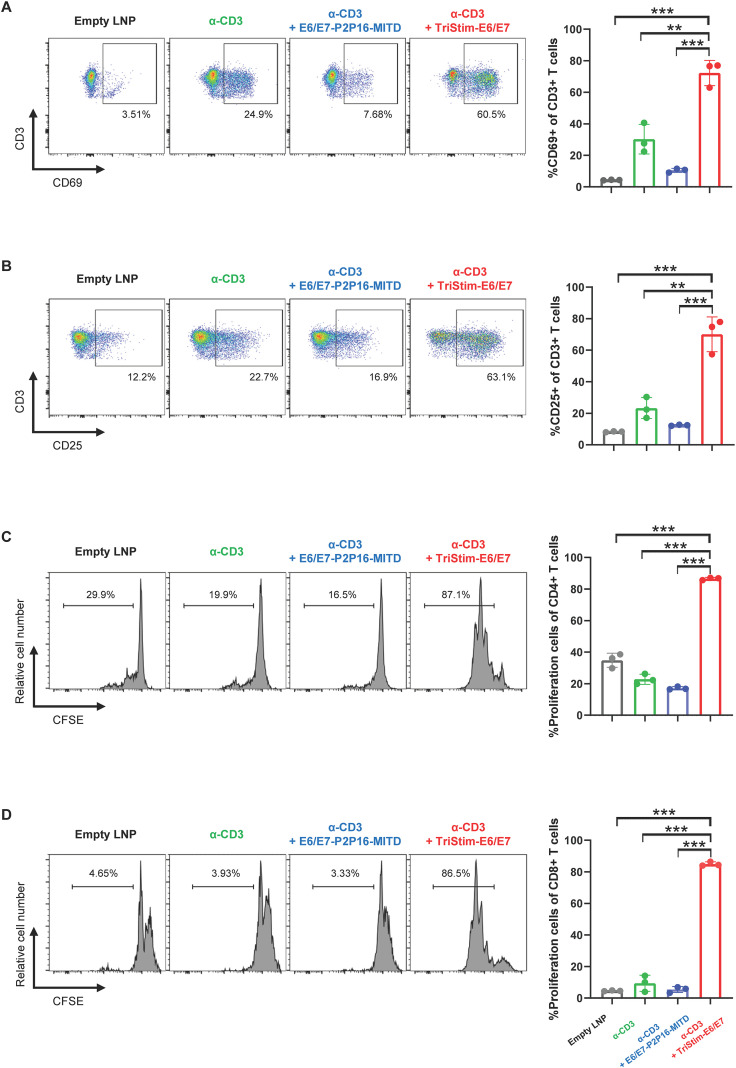
TriStim-E6/E7 mRNA induced T cell activation and proliferation *in vitro*. **(A, B)** HEK293 cells co-transfected with 0.25 μg of mb-α-CD3 mRNA and 5 μg of TriStim-E6/E7 mRNA, were used to stimulate mouse splenocytes. The expression of CD69 **(A)** and CD25 **(B)** in splenic T cells was assessed by flow cytometry analysis. Control groups included HEK293 cells transfected with empty LNPs, 0.25 μg of mb-α-CD3 mRNA alone, or combination of 0.25 μg of mb-α-CD3 mRNA and 5 μg of E6/E7-P2P16-MITD mRNA. **(C, D)** HEK293 cells co-transfected with 0.25 μg of mb-α-CD3 mRNA and 5 μg of TriStim-E6/E7 mRNA, were treated with mitomycin C before co-cultured with mouse splenocytes. The proliferation of CFSE-prestained mouse splenic T cells were assessed by flow cytometry. Control groups included HEK293 cells transfected with empty LNPs, 0.25 μg of mb-α-CD3 mRNA alone, or combination of 0.25 μg of mb-α-CD3 mRNA and 5 μg of E6/E7-P2P16-MITD mRNA. Data are presented as the mean ± standard deviation (n = 3). Statistical analysis was conducted using an unpaired, two-tailed Student’s t-test; **P ≤ 0.01, ***P ≤ 0.001.

Stimulation with HEK293 cells transfected with mb-α-CD3 alone led to mild upregulation of the T cell activation markers CD69 (**Figure 7A**) and CD25 (**Figure 7B**). However, when stimulated with HEK293 cells co-transfected with mb-α-CD3 and TriStim-E6/E7 mRNA (expressing CD80, 4-1BBL, and CD70), there was a significant augmentation of CD69 and CD25 expression in splenic T cells (**Figures 7A, B**). E6/E7-P2P16-MITD mRNA, encoding E6/E7 fused with tetanus toxoid P2P16 and MITD, was used as a negative control. This construct did not enhance T cell activation beyond that induced by mb-α-CD3 alone.

In the T cell proliferation assay, splenocytes stained with CFSE were co-cultured with HEK293 cells transfected with mb-α-CD3 and either TriStim-E6/E7 mRNA or E6/E7-P2P16-MITD mRNA. Results demonstrated that TriStim-E6/E7 mRNA-transfected HEK293 cells significantly enhanced the proliferation of both CD4^+^ (**Figure 7C**) and CD8^+^ (**Figure 7D**) T cells induced by mb-α-CD3, while E6/E7-P2P16-MITD mRNA-transfected cells did not.

Collectively, these data confirm that TriStim-E6/E7 mRNA effectively induces T cell activation and proliferation through its encoded co-stimulatory molecules.

### 3.7 TriStim-E6/E7 mRNA synergized with immune checkpoint blockade

**Figure 8:**
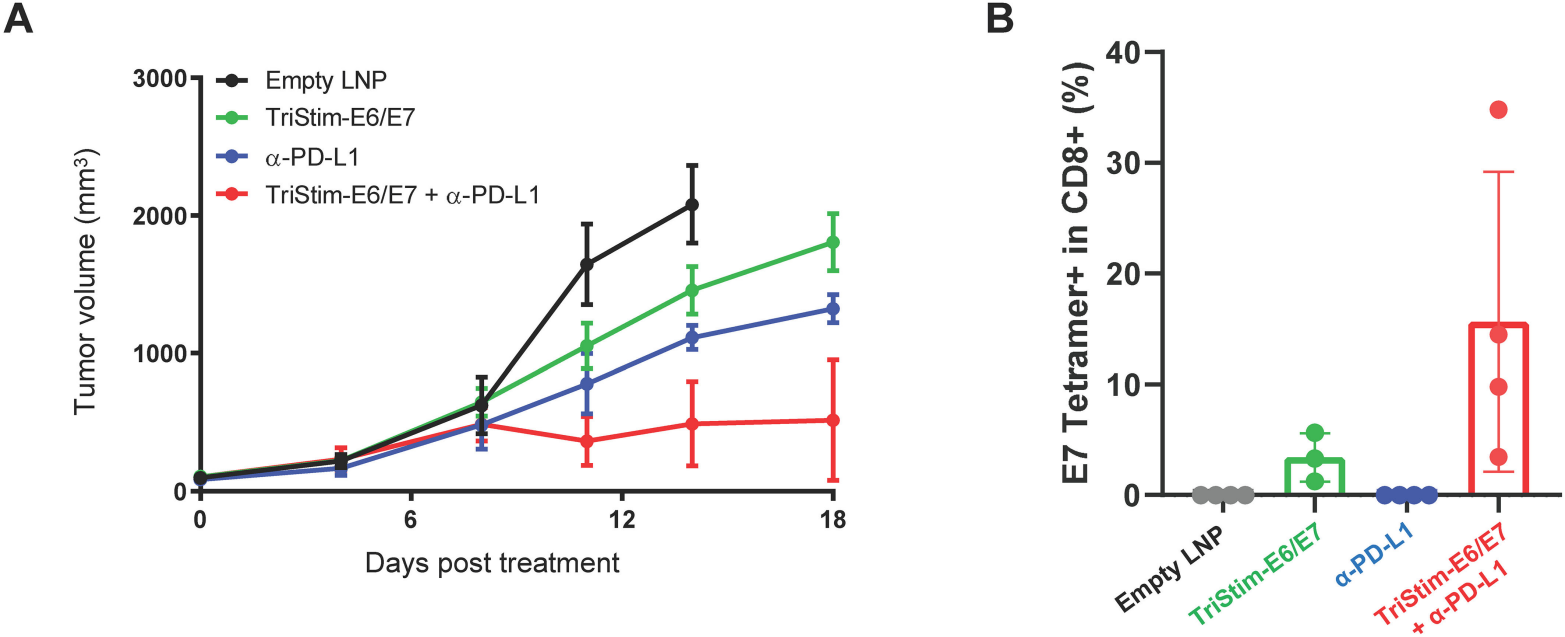
Combination treatment with TriStim-E6/E7 mRNA and immune checkpoint inhibitors improved anti-tumor efficacy. Mice bearing subcutaneous TC-1 tumors were treated with 3 μg of TriStim-E6/E7 mRNA once a week for 3 consecutive weeks (on day 0, 7 and 14 after grouping), and 200 μg of anti-PD-L1 monoclonal antibody twice a week for 3 consecutive weeks (on day 0, 3, 7, 10 and 14 after grouping), and their tumor volumes **(A)** were measured. E7 tetramer^+^ CD8^+^ T cells **(B)** in peripheral blood of mice were measured using flow cytometry, 18 days after the first dose. Data in **(A)** are presented as the mean ± standard error of the mean (n = 6). Data in **(B)** are presented as the mean ± standard deviation (n = 4).

Immune checkpoint blockade has been approved for cervical cancer treatment. PD-1 expression increases rapidly during T cell activation following vaccination (32). We evaluated the anti-tumor efficacy of combining TriStim-E6/E7 mRNA with anti-PD-L1 therapy. Low-dose TriStim-E6/E7 mRNA (3 μg/mouse/injection) and anti-PD-L1 antibody (200 μg/mouse/injection) each delayed tumor growth, with TGI rates of 64.3% and 44.9%, respectively, 18 days post-first dose (**Figure 8A**). Concurrent treatment with TriStim-E6/E7 mRNA and anti-PD-L1 antibody significantly inhibited tumor growth, achieving a TGI of 89.9%. This combination also increased the number of E7-specific T cells compared to TriStim-E6/E7 mRNA alone (**Figure 8B**). These findings demonstrate that TriStim-E6/E7 mRNA enhances the anti-tumor response when combined with immune checkpoint blockade, suggesting a promising therapeutic strategy for cervical cancer treatment.

### 3.8 Human TriStim-E6/E7 mRNA inhibited tumor growth in a humanized mouse model

Given the significant anti-tumor efficacy of murine TriStim-E6/E7 mRNA in syngeneic models, we developed a humanized version for clinical translation. This human TriStim-E6/E7 mRNA encodes HPV16 E6/E7 antigens along with human CD80, 4-1BBL, and CD70. Due to the low amino acid sequence similarity (less than 60%) between human and murine co-stimulatory molecules, we generated CD28/4-1BB/CD27 triple-humanized mice. These mice express chimeric receptors with human extracellular domains fused to murine transmembrane and intracellular domains, enabling response to human co-stimulatory molecules while maintaining mouse signaling pathways.

**Figure 9:**
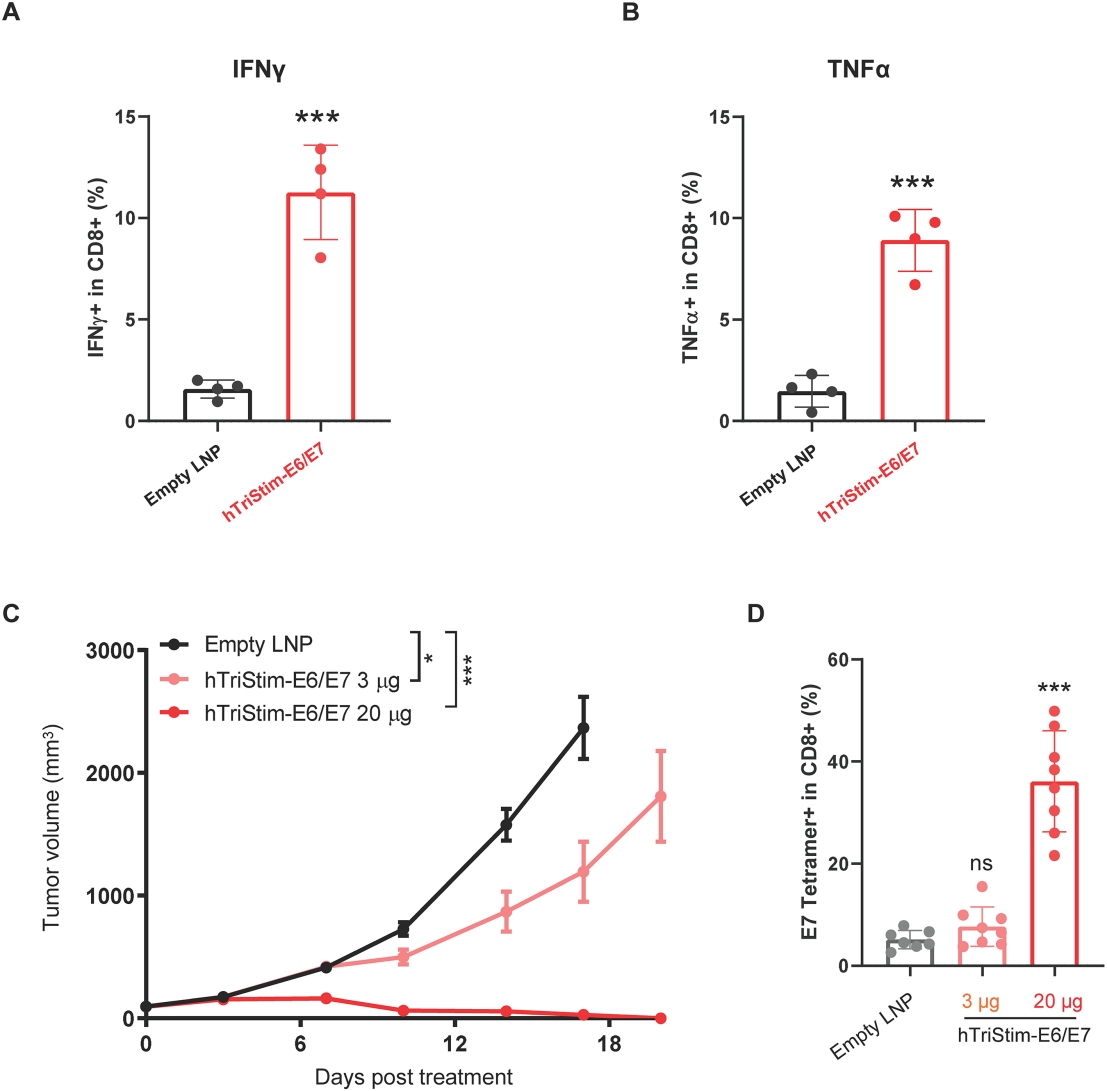
Human TriStim-E6/E7 mRNA inhibited tumor growth in a humanized mouse model. **(A, B)**. CD28/4-1BB/CD27 triple-humanized mice were intramuscularly vaccinated with 20 μg hTriStim-E6/E7 mRNA-LNP or control empty LNPs once a week for 3 consecutive weeks (on day 0, 7 and 14 after grouping), and the cellular immune responses were assessed. IFNγ-producing CD8^+^ T cells **(A)** and TNFα-producing CD8^+^ T cells **(B)** were measured using flow cytometry in splenocytes stimulated with HPV16 E6 and E7 peptide pools. **(C, D)** CD28/4-1BB/CD27 triple-humanized mice bearing subcutaneous TC-1 tumors were treated with 3 μg or 20 μg of hTriStim-E6/E7 mRNA once a week for 3 consecutive weeks (on day 0, 7 and 14 after grouping), and their tumor volumes **(C)** were measured. E7 tetramer^+^ CD8^+^ T cells **(D)** in peripheral blood of mice were measured using flow cytometry, 21 days after the first dose. Data in **(A, B)** are presented as the mean ± standard deviation (n = 4). Data in **(C)** are presented as the mean ± standard error of the mean (n = 8). Data in **(D)** are presented as the mean ± standard deviation (n = 8). Statistical analysis in **(A, B, D)** was conducted using an unpaired, two-tailed Student’s t-test; ns, not significant, *P ≤ 0.05, ***P ≤ 0.001. Statistical analysis for tumor volumes **(C)** was conducted through two-way ANOVA with Sidak’s multiple comparison test; *P ≤ 0.05, ***P ≤ 0.001.

Immunization with human TriStim-E6/E7 mRNA induced antigen-specific IFN-γ- (**Figure 9A**) and TNF-α- (**Figure 9B**) producing T cells in CD28/4-1BB/CD27 triple-humanized mice. In TC-1 tumor-bearing CD28/4-1BB/CD27 triple-humanized mice, treatment with 3 μg of human TriStim-E6/E7 mRNA significantly inhibited tumor growth, achieving a TGI of 34.8%, 20 days post first dose, while treatment with 20 μg of human TriStim-E6/E7 mRNA resulted in complete tumor eradication (**Figure 9C**). The anti-tumor activity was associated with the induction and expansion of E7-specific T cells in peripheral blood (**Figure 9D**).

These results confirm the cellular immunogenicity and *in vivo* anti-tumor efficacy of human TriStim-E6/E7 mRNA in a CD28/4-1BB/CD27 triple-humanized mouse model, supporting its potential for clinical application.

### 3.9 Human TriStim-E6/E7 mRNA did not induced cytokine release syndrome in an immune system humanized mouse model

**Figure 10:**
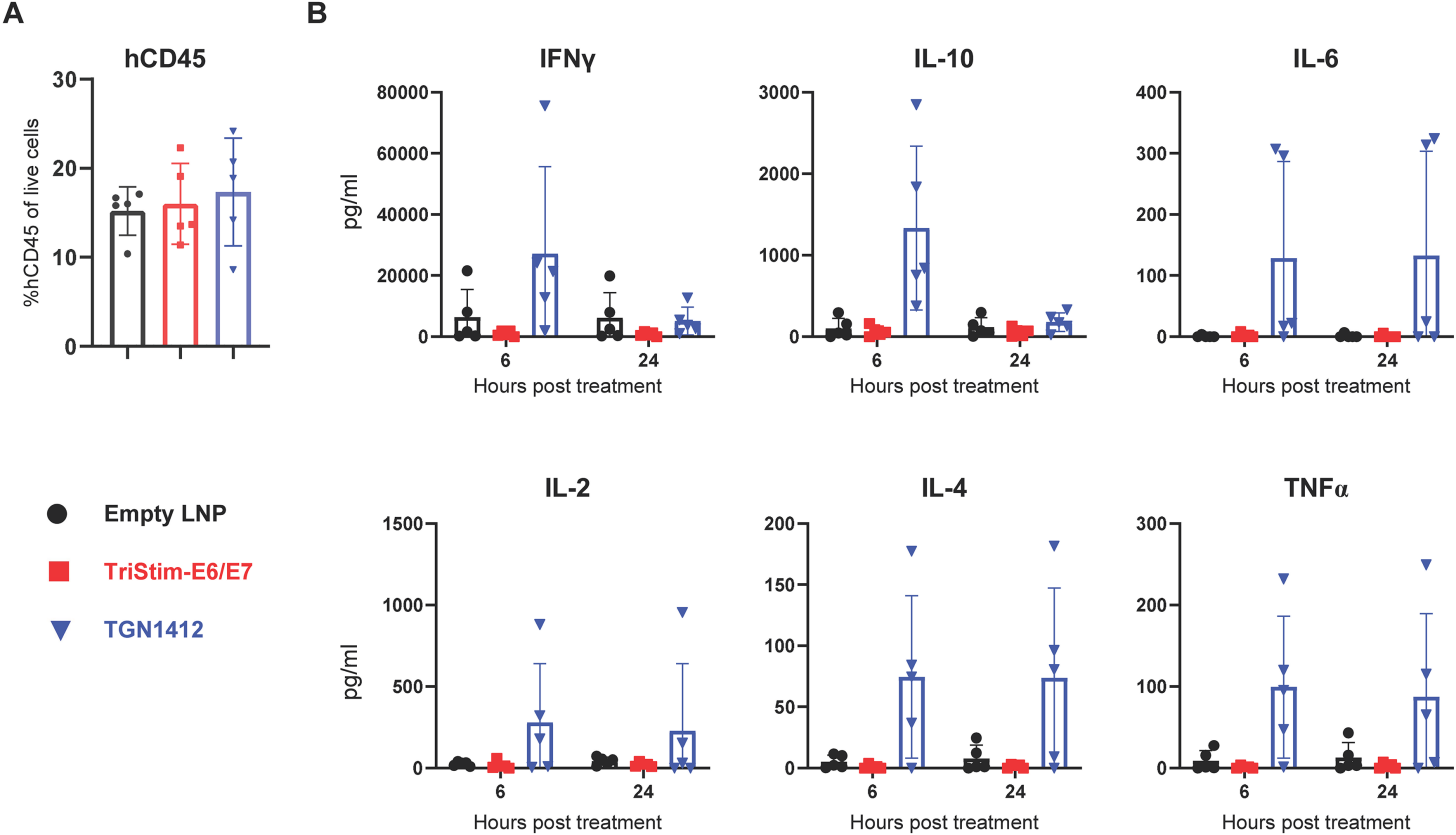
Human TriStim-E6/E7 mRNA did not induced cytokine release syndrome in an immune system humanized mouse model. **(A)** M-NSG mice were engrafted with human PBMCs. Twenty days post-engraftment, reconstitution of human leukocytes was confirmed by flow cytometry analysis of mouse peripheral blood samples using an anti-human CD45 antibody. **(B)** On day 21, PBMC-reconstituted mice were intramuscularly vaccinated with 20 μg of hTriStim-E6/E7 mRNA-LNP or control empty, or intravenously injected with 2 mg/kg of TGN1412 and their serum cytokines (IFNγ, IL-10, IL-6, IL-2, IL-4, and TNFα) were determined by cytometric bead array analysis 6 and 24 h post-treatment. Data are presented as the mean ± standard deviation (n = 5). Data are representative of two independent experiments.

Excessive T cell activation and expansion induced by immunotherapies may lead to severe adverse events, including cytokine release syndrome (CRS) (33). For instance, the CD28 superagonist TGN1412 caused life-threatening CRS in six volunteers during a Phase I clinical trial (34). To evaluate the safety profile of TriStim-E6/E7 mRNA, which encodes three T cell co-stimulatory molecules, we utilized a well-established human PBMC-reconstituted NSG mouse model (35, 36). Mice with successful engraftment (>5% human CD45^+^ cells in peripheral blood) were randomly assigned to treatment groups (**Figure 10A**). Injection of human TriStim-E6/E7 mRNA did not result in elevated levels of tested cytokines at 6 or 24 hours post-treatment compared to the LNP control group (**Figure 10B**). In contrast, TGN1412, used as a positive control, triggered significant release of IFN-γ and IL-10 at 6 hours post-injection, and moderate production of IL-6, IL-2, IL-4, and TNF-α at both 6 and 24 hours post-treatment (**Figure 10**). Unlike the CD28 superagonist, human TriStim-E6/E7 mRNA did not induce CRS in the immune system humanized mouse model, suggesting a favorable safety profile.

## 4 Discussion

Therapeutic cancer vaccines target tumor antigens and evoke host immunity against cancer. However, their clinical efficacies are limited due to pre-existed immune tolerance and immunosuppressive tumor microenvironment. In this study, we developed a novel HPV mRNA vaccine TriStim-E6/E7, which encodes E6 and E7 antigens of HPV16, as well as three T cell co-stimulatory molecules (CD80, 4-1BBL, and CD70). We systematically evaluated the effect of T cell co-stimulatory molecules on antigen immunogenicity. The results showed that TriStim-E6/E7 mRNA induced strong E7-specific cellular immune response. TriStim-E6/E7 mRNA induced robust anti-tumor efficacy and immunological memory against HPV^+^ tumor. Moreover, compared with other tested approaches of immunogenicity enhancement, TriStim-E6/E7 mRNA induced superior anti-tumor activities. Mechanistic studies revealed that expression of T cell co-stimulatory molecules significantly enhanced T cell activation and proliferation *in vitro*. Furthermore, combination of immune checkpoint inhibitors augmented the anti-tumor efficacy of TriStim-E6/E7 mRNA. For clinical translation, human TriStim-E6/E7 mRNA, which encodes E6/E7 proteins of HPV16 and three human T cell co-stimulatory molecules, elicited robust antigen-specific cellular immune response and anti-tumor efficacy in CD28/4-1BB/CD27 triple-humanized mice. Furthermore, the safety profile of human TriStim-E6/E7 mRNA was confirmed in a PBMC-reconstituted NSG mice model. Collectively, our results supported the potential application of TriStim-E6/E7 mRNA as a therapeutic vaccine for treating HPV associated malignancies.

DNA and viral vector-based therapeutic vaccines targeting E6 and E7 oncoproteins have been developed and the clinical efficacy have been validated (37–39). However, these approaches may induce deleterious genomic rearrangements and anti-viral vector immunity. In contrast, mRNA vaccines ensure transient gene expression without compromising the integrity of the host’s genomic DNA and have been validated in several pathogen vaccine development (40). Several therapeutic vaccines targeting HPV have been developed and showed promising clinical efficacy (19, 41, 42). The goal of this study is to enhance the cellular immune response and anti-tumor efficacy of therapeutic HPV vaccine by incorporating T cell co-stimulatory signaling. Our data suggested that combination of CD80, 4-1BBL and CD70 significantly augment the anti-tumor activities induced by HPV vaccine, compared with other tested T cell co-stimulatory molecule combination. In addition, this approach also has better anti-tumor efficacy compared with others, such as MITD fusion, ubiquitin fusion, and incorporating innate immune signaling.

T cell co-stimulatory molecule signaling is critical for T cell activation and memory (13). CD80, 4-1BBL, and CD70 are co-stimulatory molecules with slightly distinct functions. CD28 mediates a key co-stimulatory signal promoting T cell expansion, differentiation, and short-term survival, but CD28-CD80/CD86 interaction alone cannot fully support long-lasting T cell responses or memory T cell generation (43, 44). CD27 quantitatively enhances CD28-mediated signaling early in T cell stimulation to promote initial clonal expansion (45, 46). However, CD27 signaling may inhibit T cell responses, depending on CD27/CD70 expression level, timing, and duration—tightly regulated to ensure transient availability (47). In contrast, 4-1BB/4-1BBL signaling provides late-acting signals that inhibit activated T cell apoptosis and support effector T cell survival at primary immune response peaks (48). Thus, we hypothesize CD28, CD27, and 4-1BB act non-redundantly and synergistically in vaccine-induced immune responses, likely due to distinct signal quality and timing. In this study, we have shown that combination of CD80, 4-1BBL and CD70 significantly augments the anti-tumor activities induced by HPV vaccine, which is superior to all other tested T cell co-stimulatory molecule combination. However, how this combination modulates T cell signature and its superiority over other co-stimulatory combinations require further investigation.

One of the limitations of current study is lack of expression data of E6 protein and limited immune response to E6 antigen in mice. Despite several attempts, several commercial available antibodies against E6 protein of HPV16 fail to give positive blot in western blotting analyzing HPV16^+^ TC-1 cells or TriStim-E6/E7 mRNA transfected HEK293 cells. We can only confirm the expression of E6 protein by immunofluorescence (**Figure 1C**). Additionally, as the N terminal of 4-1BBL-E6 fusion protein, the expression of 4-1BBL was clearly demonstrated by flow cytometry. The induction of E6-specific T cells by TriStim-E6/E7 mRNA was limited, but detectable, which is line with previous studies showing limited immune response to E6 protein in C57BL/6 mice (49). In contrary, E6 elicited significant immunogenicity in Rhesus monkeys in previous study (50).

TriStim-E6/E7 mRNA elicited strong immune memory and protected mice from same tumor rechallenge. Additionally, pre-immunization with TriStim-E6/E7 mRNA prevented mice from tumor generation. This finding was supported by the observation that E7-specific T cells were significantly up-regulated upon tumor implantation in TriStim-E6/E7 mRNA immunized mice. These pre-clinical findings highlight the potential translational relevance of TriStim-E6/E7 mRNA, suggesting it may hold promise for preventing tumor recurrence in cured cervical cancer patients and blocking the progression of cervical intraepithelial neoplasia (CIN1/CIN2/CIN3) to invasive cervical malignancy. Notably, since all data are obtained from mouse models, *in vivo* efficacy and safety studies in large animals are required prior to clinical translation.

Immunotherapies with immune checkpoint blockade have remarkably changes the paradigm of cancer therapy and significantly improved the clinical outcome of tumor treatment. We observed the synergistic anti-tumor effect of combination treatment with anti-PD-L1 antibody and TriStim-E6/E7 mRNA in TC-1 tumor bearing mouse model. This finding is clinical relevant since immune checkpoint inhibitor has been approved for cervical cancer treatment, and combination treatment holds a promising therapeutic strategy.

For clinical application, we developed human TriStim-E6/E7 mRNA and tested its immune-generating ability and anti-tumor effects in CD28/4-1BB/CD27 triple-humanized mice. This is because human T-cell co-stimulatory molecules are unlikely to interact with mouse receptors. Even though a chimeric receptor strategy, combining human extracellular domains with mouse transmembrane and intracellular domains, was used to create the mouse model (51), the signaling and function of these chimeric receptors still need to be further validated. As these co-stimulatory molecules and their receptors mainly work in immune cells, immune system humanized mouse models could be an alternative. Commonly-used immune-deficient mouse models with human PBMC- or HSC-reconstitution have drawbacks. In the PBMC model, reconstituted immune cells are mostly T cells, with few antigen-presenting cells (52). In the HSC-reconstitution model, immune cell types can be adjusted with cytokines to mimic the human immune system (52, 53). However, critical immune-response organs like the thymus and lymph nodes are very small or undetectable in these models (54), impairing antigen presentation and T-cell development. Non-human primates (e.g., rhesus and cynomolgus macaques), with immune systems similar to humans, are ideal for vaccine development (55). Yet, unlike mice, they have diverse MHC molecules, potentially causing individual variation in immune responses to antigens (50).

In safety evaluations, TriStim-E6/E7 mRNA demonstrates good tolerance in immunocompetent mice. However, the expression pattern of T cell co-stimulatory molecule receptors, particularly CD28, differs between mice and humans, limiting the predictive value of this model for potential clinical adverse effects (56). Similarly, in non-human primates, CD28 expression on CD4^+^ effector memory T cells differs from that in humans, meaning cynomolgus macaques cannot reliably predict safety issues, as seen with the CD28 superagonist TGN1412 (57, 58). Conversely, the PBMC-reconstituted mouse model has been successfully used to simulate cytokine release storms induced by TGN1412 (35, 36). In our study, this model was employed, and no significant cytokine elevation was observed following TriStim-E6/E7 mRNA administration, indicating its favorable safety profile. Although the suitability of this model for evaluating 4-1BB and CD27-targeting drugs remains unclear, clinical data on 4-1BB and CD27 agonists suggest their safety in humans (59–61).

## 5 Conclusions

In summary, we have developed a novel mRNA-based therapeutic vaccine, TriStim-E6/E7, targeting the oncogenic E6 and E7 proteins of HPV16. Through comprehensive preclinical evaluation, we have demonstrated its robust immunogenicity, significant anti-tumor efficacy, and favorable safety profile. These findings collectively support the potential translation of TriStim-E6/E7 mRNA into clinical application as a therapeutic vaccine for HPV-related malignancies.

## Data Availability

The datasets presented in this article are not readily available because data are included in the article. Further inquiries can be directed to the corresponding author. Requests to access the datasets should be directed to zhigang.li@aimbio.com.
